# Line-probe assay and molecular typing reveal a potential drug resistant clone of *Mycobacterium tuberculosis* in Ethiopia

**DOI:** 10.1186/s40794-018-0075-3

**Published:** 2018-12-04

**Authors:** Shiferaw Bekele, Yohannes Derese, Elena Hailu, Adane Mihret, Kifle Dagne, Lawrence Yamuah, Tsegaye Hailu, Samuel Ayele, Demissew Beyene, Stefan Berg, Abraham Aseffa

**Affiliations:** 1 Armauer Hansen Research Institute, Jima Road, Addis Ababa, Ethiopia; 2 Department of Microbial, Cellular and Molecular Biology, College of Natural Sciences, Addis Ababa University, Addis Ababa, Ethiopia; 3 Animal and Plant Health Agency, New Haw, Surrey, UK; 4 Present address: J. Craig Venter Institute, Rockville, MD USA

**Keywords:** Mycobacterium, Tuberculosis, Drug resistance, Rifampicin, Isoniazid, MDR-TB, Genotype, Pulmonary, Cervical lymphadenitis, Clone

## Abstract

**Background:**

Antimicrobial resistance is a global concern of increasing significance. Multidrug resistant tuberculosis (MDR-TB) is spreading worldwide. It is important to monitor trends of antimycobacterial resistance. This is particularly true for high TB burden countries such as Ethiopia where disproportionally less drug sensitivity data are reported from.

**Methods:**

The prevalence of drug resistance was assessed with the line probe assay GenoType MTBDR*plus* in a set of 161 *M. tuberculosis* strains that were selected from four common lineages and sub-lineages previously identified in Ethiopia. Most of the tested *M. tuberculosis* isolates had been genotyped by established Spoligotyping and MIRU-VNTR typing methods.

**Results:**

The proportion of MDR-TB among the isolates was 3.1%. Mono-resistance was 1.2% to rifampicin and 4.3% to isoniazid, and resistance to either of the two first line drugs was 8.7%. Strains of Lineage 4 had the highest resistance rate (13.6%) followed by Lineage 3 (4.9%). None of the isolates representing Lineages 1 and Lineage 7 were drug resistant. Multidrug resistance among pulmonary TB and TB lymphadenitis clinical isolates was 2.8 and 3.7%, respectively. Drug resistance of strains carrying the most prevalent spoligotype in Ethiopia - SIT149 - was further explored. Stratification by MIRU-VNTR identified one genotype with a high rate of drug resistance against Rifampicin and Isoniazid and circulation of a potential MDR-TB clone is proposed.

**Conclusion:**

Although the strain selection was not fully randomized, the overall *M. tuberculosis* drug resistance rate in this strain set was 8.7% while the rate of MDR was 3.1%. In parallel, we identified a sub-lineage that showed a high rate of resistance to both rifampicin and isoniazid. These resistant strains may belong to a clone of *M. tuberculosis* that is circulating in the highlands of Ethiopia.

**Electronic supplementary material:**

The online version of this article (10.1186/s40794-018-0075-3) contains supplementary material, which is available to authorized users.

## Background

Multi-drug resistant *Mycobacterium tuberculosis* (MDR-TB) (resistant to at least both isoniazid and rifampicin) is an increasing threat to all efforts to bring down the prevalence of tuberculosis (TB) worldwide through control strategies such as the Directly Observed Treatment, Short-Course (DOTS) program. Recent numbers from the WHO suggest that MDR-TB can be as high as 5% globally, is present in most affected countries, and is more prevalent among HIV-infected patients [1]. Although resistant strains have been suggested to lose fitness and thus be less transmissible than strains susceptible to TB drugs [2], MDR-TB is on the rise globally and is contributing to higher mortality rates from the disease [3]. MDR-TB strains can be identified with culture on drug containing selective media as well as through molecular typing such as with Line probe assays (that recognize specific chromosomal mutations) [4]. Molecular typing techniques on culture isolates allow to pinpoint specific mutations causing resistance in *M. tuberculosis* for both therapeutic guidance and surveillance of drug resistance patterns [5]. In addition, epidemiological information on transmission routes and relative prevalence of circulating *M. tuberculosis* lineages and sub-lineages in a defined geographic region can be generated through such DNA-based typing techniques.

World-wide genotyping efforts have revealed seven major *M. tuberculosis* lineages (Lineages 1–7) that are established in humans of which two, Lineages 5 and 6, were more commonly known as *Mycobacterium africanum*. The geographical distribution varies between these lineages; Lineage 4 (L4) is the dominating lineage in Europe and the Americas while Lineages 1, L2 and L3 are more prevalent in Africa and Asia. Interestingly, *M. africanum* Lineages 5 and L6 are only limited to West Africa while recent epidemiological work suggests that *M. tuberculosis* Lineage 7 is restricted to Ethiopia [6]. From an evolutionary point of view, it is interesting to note that five out of these seven lineages have been identified in Ethiopia (L1-L4, L7) suggesting a long history of TB in the country [6–8].

Both pulmonary tuberculosis (PTB) and extra-pulmonary tuberculosis (EPTB) are significant public health problems in Ethiopia [9]. EPTB is clinically diagnosed or bacteriologically confirmed in organs other than the lungs. Tuberculous lymphadenitis (TBLN) is the most commonly occurring form of EPTB. It manifests as a slowly progressive, painless swelling of lymph nodes, and is caused by strains that are also isolated from cases of PTB including those that are drug resistant [10].

In Ethiopia, comparisons of drug resistance have not been well investigated across the main lineages. The present study was therefore conducted to explore and compare drug resistance patterns among the most prevalent *M. tuberculosis* lineages in Ethiopia including the newly identified L7, using isolates from both PTB and TBLN cases. We analyzed a previously published collection of *M. tuberculosis* strains from Ethiopia [11] to get insight into drug resistance and MDR-TB rates among the selected isolates.

## Methods

### Strain selection

One hundred and sixty-one *M. tuberculosis* strains used for this study were selected from four lineages from 950 archived isolates that were collected in a cross sectional study during 2006–2010 conducted to identify the disease agents causing PTB and TBLN in Ethiopia [11]. The representation of geographical origins and genotypes with regards to the original study [11] are shown in Additional file 1: Tables S1, Additional file 2: Table S2 and Additional file 3: Table S3. All 950 isolates had previously been characterized as *M. tuberculosis* [11] by RD (Region of Difference) typing and Spoligotyping [12, 13] and a subset of these strains had been defined [11] by MIRU-VNTR typing [14].

### Drug susceptibility testing

To explore drug susceptibility among *M. tuberculosis* isolates, the Line-probe assay Genotype MTBDR*plus* (Hain Lifescience, Germany) was performed according to protocol by manufacturer [15]. This assay is designed to detect strains of the *M. tuberculosis* complex from clinical specimens and culture, and whether they are resistant to RMP and/or INH.

### Data collection

The data collected by Firdessa et al. 2013 [11] was the baseline data for this work and formulated in Microsoft Access. Data was analyzed using STATA version 11 (Stata Statistical Software: College Station, TX: StataCorp LP).

## Results

### Strain selection

Drug susceptibility testing was undertaken on 161 strains selected from 950 previously characterized and archived *M. tuberculosis* strains originally isolated from patients in 9 hospitals/health centers in a comprehensive Ethiopian cross sectional study [11]. At large, these 161 *M. tuberculosis* strains, isolated from 54 TBLN and 107 PTB patients, represented the most common spoligotype(s) from each of four different lineages found in that study and they are listed in the Additional file 1: Table S1. In particular, 41 isolates of SIT25 (L3) and 88 isolates from SIT149 (L4) - the two most frequent spoligotypes in that study - were randomly selected among these two types from the archived strain pool. The percentage of sampled isolates for this study among the total archive from the previously reported survey [11] was 7/11 (63%) for L1, 41/252 (16%) for L3, 88/671 (13%) for L4, and 25/36 (69%) for L7.

### Line probe assay

All 161 selected isolates were heat-inactivated and tested by GenoType MTBDR*plus (v1.0)* (Hain Lifescience GmbH, Germany) for Rifampicin (RMP) and Isoniazid (INH) resistance. This line probe assay identified 14 (8.7%) isolates as resistant to at least one of these two first line anti-tuberculosis drugs. The total number of isolates resistant to INH was 12/161 (7.5%). The proportion of RMP resistant strains was 4.3% (7/161) while MDR was detected in 3.1% (5/161) of cases (Table 1). When stratified by disease type, mono-resistance was observed in 3.7% (4/107) of PTB and 7.4% (4/54) of TBLN cases. MDR was detected in 3/107 (2.8%) PTB and 2/54 (3.7%) TBLN isolates. When drug resistance was compared among lineages, a higher proportion was observed among L4 strains where 5/88 (5.7%) were MDR and 7/88 (8.0%) mono-resistant compared to L3 where there was no MDR strain and only 2/41 (4.9%) were mono-resistant (Table 1). The number of isolates representing L1 and L7 was low; nevertheless, none of the seven L1 strains and the 25 strains of L7 was resistant to any of the drugs tested.

**Table 1:** Drug resistance of *Mycobacterium tuberculosis* isolates stratified by lineage, Ethiopia^a^

Lineage	Number of isolates	Susceptible (%)	Resistant only to RMP (%)	Resistant only to INH (%)	Resistant to both RMP and INH (MDR) (%)
L1	7	7 (100)	0	0	0
L3	41	39 (95.2)	1 (2.4)	1 (2.4)	0
L4	88	76 (86.4)	1 (1.1)	6 (6.8)	5 (5.7)
L7	25	25 (100)	0	0	0
All	161	147 (91.4)	2 (1.2)	7 (4.3)	5 (3.1)

^a^Drug sensitivity was determined with the line probe assay GenoType MTBDR*plus*

### Drug resistance among SIT149 genotypes

Most (12/14) of the drug resistant *M. tuberculosis* isolates identified by the line probe assay in this study belonged to the subset of L4 strains with spoligotype SIT149. These 12 strains come from a subset of 88 SIT149 strains that had previously been characterized by MIRU-VNTR as well [11] leading to further stratification into 24 different genotypes named SIT149:A-Y as shown in Additional file 1: Table S1. Superimposing the genotyping data and the drug resistance results of these 88 isolates showed that the 12 drug resistant strains were clustered to only six genotypes; five strains belonged to SIT149:A, three were of SIT149:L, while the remaining four strains were of genotypes SIT149:B, C, I, and U. In fact, all five strains genotyped as SIT149:A were drug resistant according to the line probe assay (Additional file 1: Table S1), and four of these five strains were actually resistant to both RMP and INH with identical mutation profiles (Table 2); RMP resistance confirmed by mutation “MUT3” (*rpoB* codon S531 L) and INH resistance by mutation “MUT1” (C15T upstream *katG*). In comparison, the three drug resistant isolates of genotype SIT149:L (which only diverges from SIT149:A in the number of repeats in MIRU-VNTR locus 2401) showed different mutation profiles (Table 2).

**Table 2:** Demographic (A), genotyping (B), and drug resistance (C) data of eight *Mycobacterium tuberculosis* strains of genotypes SIT149:A and SIT149:L

A.	Sample ID	BTBH003	BTBS101	BTBS119	BTBH455	BTBS785	BTBS341	BTBS571	BTBH946
	Site	Woldiya	Gondar	Fiche	Woldiya	Fiche	Butajira	Woldiya	Butajira
	Isolation year	2006	2009	2009	2007	2010	2009	2010	2009
B.	Genotype^a^	A	A	A	A	A	L	L	L
	580	2	2	2	2	2	2	2	2
	2996	1	1	1	1	1	1	1	1
	802	2	2	2	2	2	2	2	2
	960	5	5	5	5	5	5	5	5
	1644	1	1	1	1	1	1	1	1
	3192	3	3	3	3	3	3	3	3
	424	1	1	1	1	1	1	1	1
	577	4	4	4	4	4	4	4	4
	2165	3	3	3	3	3	3	3	3
	*2401*	5	5	5	5	5	4	4	4
	3690	3	3	3	3	3	3	3	3
	4156	3	3	3	3	3	3	3	3
	2163b	1	1	1	1	1	1	1	1
	1955	3	3	3	3	3	3	3	3
	4052	7	7	7	7	7	7	7	7
	154	2	2	2	2	2	2	2	2
	2531	5	5	5	5	5	5	5	5
	4348	2	2	2	2	2	2	2	2
	2059	2	2	2	2	2	2	2	2
	2687	1	1	1	1	1	1	1	1
	3007	3	3	3	3	3	3	3	3
	2347	4	4	4	4	4	4	4	4
	2461	2	2	2	2	2	2	2	2
	3171	3	3	3	3	3	3	3	3
C.	RMP^b^	R	R	S	R	R	S	R	S
	INH^b^	R	R	R	R	R	R	R	R
	rpoB WT1	+	+	+	+	+	+	+	+
	rpoB WT2	+	+	+	+	+	+	+	+
	rpoB WT3	+	+	+	+	+	+	+	+
	rpoB WT4	+	+	+	+	+	+	+	+
	rpoB WT5	+	+	+	+	+	+	+	+
	rpoB WT6	+	+	+	+	+	+	+	+
	rpoB WT7	+	+	+	+	+	+	+^c^	+
	*rpoB WT8*	–	–	+	–	–	+	+^c^	+
	rpoB Mut1	–	–	–	–	–	–	–	–
	*rpoB Mut2A*	–	–	–	–	–	–	+	–
	*rpoB Mut2B*	–	–	–	–	–	–	+	–
	*rpoB Mut3*	+	+	–	+	+	–	+	–
	*inhA WT1*	+	+	+	+	+	–	+^c^	–
	inhA WT2	+	+	+	+	+	+	+^c^	+
	*inhA Mut1*	–	–	–	–	–	+	–	+
	*inhA Mut2*	–	–	–	–	–	–	+	–
	inhA Mut3A	–	–	–	–	–	–	–	–
	*inhA Mut3B*	–	–	–	–	–	–	+	–
	katG WT	–	–	–	–	–	–	–	–
	*katG Mut1*	+	+	+	+	+	–	–	–
	katG Mut 2	–	–	–	–	–	–	–	–

Loci with variability between isolates with regards to genotype and drug resistance are underlined and in *italic*

^a^Genotype refers to Spoligotype SIT149 stratified by number of repeats in respective MIRU-VNTR locus shown here (580; 2996; 802; 960; 1644; 3192; 424; 577; 2165; 2401; 3690; 4156; 2163b; 1955; 4052; 154; 2531; 4348; 2059; 2687; 3007; 2347; 2461; 3171). A, genotype SIT149:A; L, genotype SIT149:L

^b^*S* drug susceptible, *R* drug resistant, + positive hybridization, − negative hybridisation

^c^Positive hybridisation of wild type probe despite hybridisation to a corresponding mutation; indication of dual infection or cross-contamination of sample

### Geographical distribution of SIT149 genotypes

**Fig. 1:**
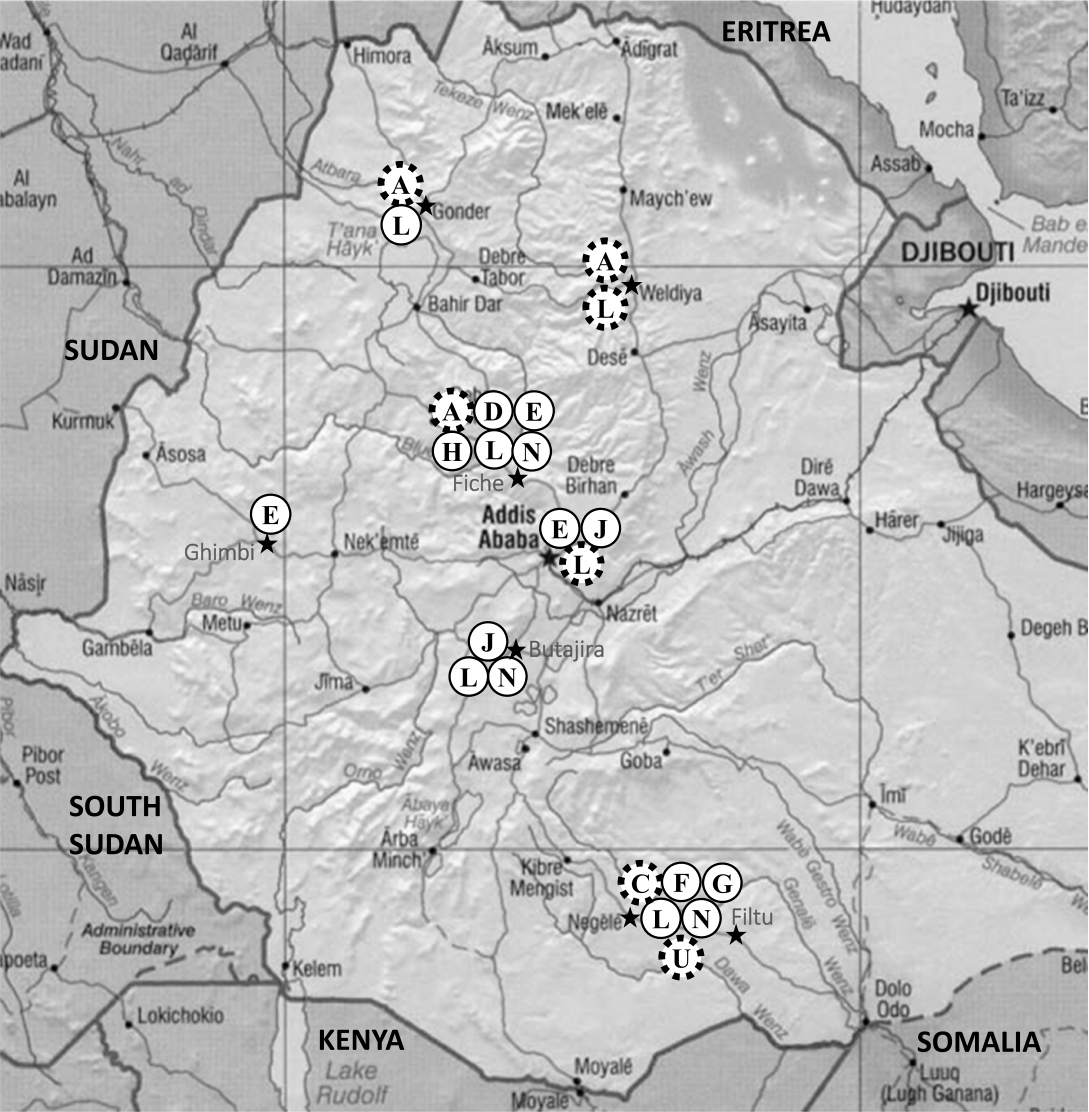
Geographical distribution of selected genotypes of *Mycobacterium tuberculosis* shared spoligotype SIT149 in Ethiopia as identified by Firdessa et al. [11]. Study site is marked with a black star. Capitalized letters symbolize genotype of SIT149. Only genotypes with two or more isolates are shown (Additional file 1: Table S1). Dotted line symbolizes genotype with at least one drug resistant isolate identified in that study site

To get a picture of the geographical distribution of the identified SIT149 genotypes, we mapped all genotypes with two or more *M. tuberculosis* isolates (Additional file 1: Table S1; Fig. 1). The most prevalent genotype SIT149:L, which included 31 of 88 strains of spoligotype SIT149, was isolated from patients in most study sites while the other genotypes of SIT149 showed different degrees of geographical clustering. Interestingly, genotype SIT149:A that was shown in this study to have a high rate of drug resistance was only found in Woldiya, Gondar, and Fiche in the Central and Northern Ethiopian highlands. All other identified genotypes were isolated from Central or Southern Ethiopia.

## Discussion

Ethiopia has a long history of human TB [6] and is still hugely affected by the disease and is listed among the TB high-burden countries in the world, with an estimated number of 172,000 new cases (an incidence rate of 164/100,000) in 2017 [1]. Since the introduction of anti-TB drugs in the 1940s, and especially since the 1990s, drug-resistant TB has become a significant threat in the overall fight against TB. For 2017 WHO reported an MDR-TB incidence rate among new TB cases of 3.7% worldwide and 2.7% for Ethiopia, i.e. 5500 new cases per year [1].

In the present study, we investigated about 17% of 950 strains that were collected in one of the largest molecular epidemiology studies of TB in Ethiopia [11]. *M. tuberculosis* isolates of all four lineages that had been identified were selected with particular focus on the two most prevalent spoligotypes, SIT149 (L4) and SIT25 (L3), as well as strains of the recently identified L7. Here we report an overall drug-resistance rate of 8.7% and an MDR-TB rate of 3.1% among these strains. A limitation of the study is that the initial population from which the isolates were collected, although extensive and covering several regions of the country, was not randomly selected to be representative of the national population and not all the strains identified were tested for drug resistance. Nevertheless, these figures fall within the range of WHO estimates for Ethiopia [1].

There was a marked difference in rates between lineages. Strains of L1 and L7 were all susceptible to RMP and INH but the number of isolates representing these lineages was relatively low, suggesting that representative results may not have been gained at the population level although a relatively higher proportion of samples was tested from the archived isolates for these lineages in this study. The overall prevalence of these lineages is low in the country [11]. Lineage 2, reported to have a tendency to higher frequency of drug resistance [16] is in particular rarely isolated in Ethiopia [7, 8, 11]. Modern and ancient TB lineages have been reported to differ in virulence and their tendency to acquire drug resistance [17]. It can therefore not be excluded that drug resistance is less frequent among L1 (ancient lineage) and L7, a pre-modern lineage that is rarely found outside Ethiopia [18].

The difference between SIT149 and SIT25 was also noticeable with a higher rate of resistance in SIT149. MDR-TB isolates were only found among genotypes of SIT149 in this study. SIT 149 (also referred to as T3-ETH, ETH-3 [18–20] or more recently as L4.2.ETH1 [6]) is a predominant spoligotype cluster in Ethiopia, more often associated with drug resistance than other spoligotypes in the country [21] and indeed elsewhere, as among Ethiopian immigrants in Israel [22]. Similarly, Agonafir et al. (2010) [23] found SIT149 as the most prevalent and most clustered spoligotype among 46 MDR-TB cases in Addis Ababa, followed by SIT21 and SIT25 (both considered as sub-lineages of L3). However, these observations do not necessarily indicate that these spoligotypes are more prone to be drug-resistant but could rather be a consequence of high prevalence in the population. In fact, a recent review by Panwalkar et al. (2017) [24] summarized many studies in attempts to find correlation between genotypes and TB drug-resistance, but they concluded that it is still uncertain whether there is such an association. On the other hand, it is frequently observed that certain clones emerge from different genotype families and spread rapidly in different populations. The Beijing B0/W148 cluster is a well-known example from Lineage 2 [25]. The Ural family of Lineage 4, with which SIT 149 clusters, although previously considered less transmissible, less virulent and not linked to MDR, has recently been reported to have given rise to MDR strains in Eastern Europe and Russia [26].

In an earlier study, we performed MIRU-VNTR on 88 strains of SIT149 [11] and here we have overlapped that information with the results gained from the line probe assay of the same strains. The most common genotype SIT149:L was identified with ~ 10% drug resistance (3 out of 31 isolates) of which two isolates were mono-resistant to INH and the third resistant to both INH and RMP (Table 2). In contrast, all five strains of genotype SIT149:A were drug-resistant and four of them (80%) had an identical resistance profile for INH and RMP in the line probe assay. The latter profile was different to the one found for the MDR-TB strain of SIT149:L. Although the number of isolates of this genotype is fairly low, the observation is important in that it could indicate that a potential MDR-TB clone of genotype SIT149:A is circulating in the Ethiopian highlands. Investigation of currently circulating MDR-TB strains in Ethiopia could explore if this clone is spreading.

## Conclusion

This study examined the rate of RMP and INH resistance among a set of 189 *M. tuberculosis* strains, representing the most common lineages and sub-lineages identified in Ethiopia. The overall drug resistance rate in the series of isolates tested was 8.7% while the rate of MDR was 3.1%. Although the study design does not allow for generalization since sampling was not based on randomized selection to achieve national representation, and drug resistance was determined with LPA rather than with the gold standard proportional method, the findings do provide insight into existing drug resistance trends in the country. Moreover, we have identified a sub-lineage of *M. tuberculosis* L4 that showed a high rate of resistance to both RMP and INH. These drug resistant strains may belong to a clone of *M. tuberculosis* that is circulating in the highlands of Ethiopia.

## Additional files

Additional file 1: **Table S1.** Summary of strain characteristics for 161 *Mycobacterium tuberculosis* isolates included in this study. (XLSX 28 kb)

Additional file 2: **Table S2.** Delineation of isolates used in this study. (XLSX 12 kb)

Additional file 3: **Table S3.** Strain selection in relation to previous survey. (DOCX 13 kb)

## Abbreviations

INH: Isoniazid
MDR: Multi-drug resistant
MIRU-VNTR: Mycobacterial Interspersed Repetitive Unit – Variable Number Tandem Repeat
PTB: Pulmonary TB
RMP: Rifampicin
SIT: Shared international type
TB: Tuberculosis
TBLN: TB lymphadenitis in the cervical lymph node
