# Supplementary material for: Line-probe assay and molecular typing reveal a potential drug resistant clone of Mycobacterium tuberculosis in Ethiopia
Source: Trop Dis Travel Med Vaccines. 2018 Dec 4;4:15. doi: 10.1186/s40794-018-0075-3 (PMC6280437; doi:10.1186/s40794-018-0075-3)
Supplement: Supplementary file 3 — Table S3. Strain selection in relation to previous survey. (DOCX 13 kb) [file 40794_2018_75_MOESM3_ESM.docx]

**Strain selection**

The selection within the lineages was random but the total numbers selected had different proportions as seen below. The emphasis was on comparing among the dominant spoligotypes in circulation. The geographic distribution of the strains tested for DR is similar to the distribution of isolates in the original study (shown in Supplementary Table)

| Strain lineage | Total isolates  (Original study, Ref 11) | Tested for MIRU-VNTR  (Original study, Ref 11) | Tested for drug resistance  in current study (n) | Proportion of isolates tested for DR  in current study) (%) | Proportion of spoligotypes  tested for DR  in current study |
| --- | --- | --- | --- | --- | --- |
| L1 | 11 | - | 7 | 7/11 (64) | 6/9 |
| L3 | 238 | 73 | 41* | 41/238 (17) | 1/32 |
| L3: SIT 125 | 125 | 73 | 41 | 41/125 (33) | 1/1 |
| L4 | 665 | 90 | 88* | 88/665 (13) | 1/129 |
| L4: SIT 149 | 150 | 90 | 88 | 88/150 (59) | 1/1 |
| L7 | 36 | - | 25 | 25/36 (69) | 5/7 |
| Total | 950 | 163 | 161 | 163/950 (17) | 13/177 |
|  |  |  |  |  |  |

*All the strains of L3 tested for drug sensitivity belonged to SIT 125 and all the L4 strains tested for drug sensitivity belonged to SIT 149.

The study design of the initial population from which the isolates were obtained, although extensive and covering the main geographical regions/populations in Ethiopia affected by high TB burden, it was not designed as a population-based survey stratified and randomized for representativeness. Patients were enrolled consecutively through a passive health-facility based approach. However, the strain pool and data set generated was extensive.

We have not done drug sensitivity testing on all the strains identified in the large survey due to logistic challenges.

The report focuses on the dominant SITs in the main lineages of L3 and L4 (but covered most of the spoligotypes in the less prevalent L1 and L7 where we had tested about 2/3 of the isolates we had in total).
